# Fecal Incontinence in Inflammatory Bowel Disease (IBD): Associated Factors and Impact on the Quality of Life of Patients in an IBD Clinic in Switzerland

**DOI:** 10.7759/cureus.90248

**Published:** 2025-08-16

**Authors:** Perle O Hammond, Jose Diego Marques Santos, Jacob Alhassan, Christoph Matter, Frank W Seibold

**Affiliations:** 1 Gastroenterology, Intesto, Crohn Colitis Center, Bern, CHE; 2 Gastroenterology and Hepatology, Yverdon Center for Gastroenterology and Endoscopy (CYGNE), Yverdon, CHE; 3 Medicine, University of Saskatchewan, Saskatoon, CAN

**Keywords:** fecal incontinence, inflammatory bowel disease, prevalence, quality of life, risk factors

## Abstract

Background: The prevalence of fecal incontinence (FI) among patients with inflammatory bowel disease (IBD) and its associated factors is not well-studied. This research estimated the prevalence of FI in Swiss IBD patients, identified risk factors, and assessed its relationship with well-being, anxiety, and depression.

Methods: A cross-sectional study was conducted on adult IBD patients at a Swiss clinic in Bern and Fribourg. The study excluded individuals with a stoma, J-pouch, or unspecified IBD. The survey included 53 questions on demographic and clinical variables. Logistic regression was used to calculate odds ratios (ORs) with 95% confidence intervals (CIs).

Results: Among 392 respondents, 116 patients (29.5%) reported FI in the previous four weeks. FI was associated with age > 50 years (OR 2.74), vaginal delivery (OR 2.49), disease duration > 15 years (OR 2.04), abdominal pain (OR 2.56), diarrhea (OR 3.70), blood in stool (OR 3.33), >3 bowel movements/day (OR 4.50), nighttime bowel movements (OR 3.98), simple clinical colitis activity index (SCCAI) ≥ 3 (OR 6.06), extraintestinal manifestations (EIMs) (OR 1.64), and a history of anal fistula (OR 2.80). FI was significantly associated with poor well-being (OR 2.64), depression (OR 2.43), and anxiety (OR 1.88).

Conclusion: FI affects nearly one in three IBD patients and is independently associated with several disease-related factors including a history of anal fistula and the presence of EIM, which is newly described in the literature. FI negatively impacted quality of life, increasing anxiety and depression. These findings emphasize the importance of a comprehensive, patient-centered approach to identify and address risk factors promptly.

## Introduction

Inflammatory bowel disease (IBD) is a chronic condition associated with a high burden of physical and psychological symptoms [1]. Among these, fecal incontinence (FI)-the involuntary loss of stool or gas-remains underrecognized, despite its substantial impact on patients’ quality of life (QoL) and mental health [2,3]. Prevalence estimates of FI in IBD vary widely across studies, ranging from 20% to over 73%, depending on the disease subtype, definition, and methodology [4].

FI is associated with significant psychosocial distress. Patients often report feelings of shame, embarrassment, and powerlessness. The fear of incontinence may lead to heightened anxiety, reduced self-esteem, and social withdrawal, thereby further impairing emotional well-being [2,3,5].

In addition to its personal impact, FI imposes a notable burden on healthcare systems. Unrecognized and unmanaged symptoms can contribute to increased outpatient visits, diagnostics, and healthcare costs. Early detection and management of FI in IBD patients are crucial to lessen financial strain and improve QoL, alleviating personal and systemic healthcare challenges [6,7].

Despite growing recognition of FI’s importance, most prior studies lack detailed patient-reported outcomes and often omit assessments of QoL and mental health [3]. FI has been directly associated with older age [3,8,9], runny stools [8,9], and higher disease activity and duration in the IBD population [3,8,10]. A gap still exists in the IBD literature concerning FI, particularly regarding its prevalence, predictive factors, effects on QoL, and treatment options [11].

A clearer understanding of how common FI is and what causes it, as well as its significant impact on QoL, may encourage gastroenterologists to recognize its importance for IBD patients, leading to more targeted evaluations and treatments. This study aimed to (1) find out how common FI is in a group of Swiss IBD patients, (2) identify related clinical and mental health risk factors, and (3) examine how FI affects overall well-being, as well as self-reported anxiety and depression levels.

## Materials and methods

Study design and population

We conducted a multicenter, cross-sectional study between August and November 2023 at our IBD center, which operates on two sites (Bern and Fribourg). These centers serve over 1,000 patients and are staffed by certified IBD specialists and nurses trained in IBD care. The study aimed to assess the prevalence of FI, identify associated risk factors, and evaluate its impact on QoL.

Adult patients (≥18 years) with a confirmed diagnosis of Crohn’s disease (CD) or ulcerative colitis (UC) were eligible. Exclusion criteria included prior colectomy with stoma, J-pouch surgery, unclassified IBD, and incomplete survey data.

Survey procedure

The survey was available in French and German (Supplemental materials 1, 2). Study nurses distributed a 53-item paper questionnaire in person to all patients attending routine appointments at either site. Participation was voluntary and anonymous. We emphasized that patient care would remain unaffected. Patients completed the survey privately (in waiting areas or infusion rooms) and submitted it to a secure collection box. A study nurse was available to clarify any questions.

Measures

The survey had 53 questions that looked at basic information about the participants (age, sex, type of disease, how long they had it, smoking habits, whether they had a vaginal delivery, rectum involvement, and peri-anal disease) and how severe the disease was based on the short CD activity index (sCDAI) [12] for CD and the simple clinical colitis activity index (SCCAI) for UC [13]. FI was reported by the patients themselves, based on any involuntary loss of stools in the previous four weeks, and its severity was measured using the Wexner and Vaizey scales, which are recognized for their accuracy. Urgency was assessed by checking if patients could wait more than 15 minutes to go to the bathroom and by using a scale where patients rated their urgency over the last 24 hours from 0 (no urgency) to 10 (worst urgency) [14,15]. Higher scores reflect greater urgency severity, indicating a stronger immediate need for a bowel movement [16]. QoL was evaluated with the World Health Organization (WHO)-5 well-being index [17]. We also evaluated self-reported levels of anxiety and depression alongside FI-focused concerns.

Statistical analysis

Statistical analysis was performed using SPSS software (IBM Corp., Armonk, NY, US). Descriptive statistics were used to summarize patient characteristics. Associations between clinical factors and FI were assessed using logistic regression models. Both crude and adjusted odds ratios (ORs) were reported with 95% confidence intervals (CIs). Multivariable models were adjusted for age, sex, and disease duration.

## Results

Of 450 questionnaires distributed, 421 were returned (93.5% response rate). After excluding 29 responses, 12 had missing data, six had a current stoma, four had J-pouches, and seven had unspecified IBD; 392 valid responses were analyzed.

Patients’ characteristics

The cohort consisted of 194 men (49.5%) and 198 women (50.5%), with a mean age of 43.3 years (range: 18-89 years). Of the participants, 246 (64.1%) were diagnosed with CD, while 138 (35.9%) had UC. Most participants (58.4%) had a disease duration of less than 15 years and were non-smokers (77.8%) (Table 1).

**Table 1:** Demographic and clinical characteristics of IBD patients in the study cohort IBD: inflammatory bowel disease; EIM: extraintestinal manifestation; UC: ulcerative colitis; CD: Crohn's disease *Analysis for women only

Variable		Total = 392 (100%)
Age	Mean (Std dev)	43.33 (15.36)
	≤50 years	270 (68.9%)
	>50 years	122 (31.1%)
Sex	Male	194 (49.5%)
	Female	198 (50.5%)
Vaginal delivery (♀)*	Never	127 (66.1%)
	Once or more	65 (33.9%)
Smoker	Yes	87 (22.2%)
	No	305 (77.8%)
Disease type	UC	138 (35.9%)
	CD	246 (64.1%)
Disease duration	≤15 years	229 (58.4%)
	>15 years	163 (41.6%)
Rectum involved	Yes	195 (50.3%)
	No	193 (49.7%)
History of the anal fistula	Yes	85 (21.9%)
	No	304 (78.1%)
Active anal fistula	Yes	21 (5.3%)
History of or active seton drainage	Yes	59 (15.1%)
Self-reported flares during the current year	None	260 (66.3%)
	1 or more	132 (33.7%)
Treatment with biologic(s)	Yes	358 (91.3%)
	No	34 (8.7%)
Number of therapies	0 to 1	146 (37.3%)
	2 or more	245 (62.7%)
Number of EIM(s)	0	260 (66.3%)
	1 or more	132 (33.7%)

Disease characteristics and activity

In our cohort, 50.3% self-reported rectal involvement, and 21.9% reported a history of anal fistula. One-third (33.7%) experienced flares in the past year. The majority of patients (91.3%) were receiving biologic therapies. One-third of respondents (33.7%) reported extraintestinal manifestations (EIMs), primarily arthropathy (27.3%).

According to the sCDAI scores, 84.1% of the CD patients were in remission (with an sCDAI score of less than 150), while 15.9% had active disease. About 67.9% of the UC patients were in remission (SCCAI scores of 0 to 2), and the rest had active disease (SCCAI scores of 3 or higher).

FI prevalence and severity

Close to one-third of participants who reported FI reported that it happened “sometimes” (29.3%), while 11.2% reported experiencing FI weekly and 5% daily (Table 2).

**Table 2:** Frequency of FI episodes in UC and CD patients FI: fecal incontinence; UC: ulcerative colitis; CD: Crohn’s disease; “rarely”: 1 episode in the past 4 weeks; “sometimes”: >1 episode in the last 4 weeks, but <1 episode weekly; “weekly”: 1 or more episodes weekly, but <1 episode daily; “daily”: 1 or more episodes daily

Frequency of FI	UC n = 42, n (%)	CD n = 74, n ( %)	Total n = 116, n (%)
Rarely	24 (57.1%)	38 (51.4%)	62 (53.4%)
Sometimes	10 (23.8%)	24 (32.4%)	34 (29.3%)
Weekly	5 (11.9%)	8 (10.8%)	13 (11.2%)
Daily	3 (7.1%)	4 (5.4%)	7 (6%)

Among those with FI, 53.4% reported only one episode, and 75.2% identified liquid stool as the primary trigger. Notably, 26% experienced incontinence with solid stool, highlighting variability in symptom severity (Table 3).

**Table 3:** Severity of fecal incontinence in inflammatory bowel disease (IBD) patients, assessed by Vaizey & Wexner scores *Analysis for females only “Rarely”: 1 episode in the past 4 weeks; “sometimes”: >1 episode in the last 4 weeks, but <1 episode weekly; “weekly”: 1 or more episodes weekly, but <1 episode daily; “daily”: 1 or more episodes daily 1: Vaizey & Wexner items; 1*: Vaizey: bivariate yes or no; 2: Wexner only; 3: additional analysis Vaizey: minimum score: 0: perfect continence; maximum score: 24: complete incontinence Wexner: minimum score: 0: perfect continence; maximum score: 20: complete incontinence

Incontinence type	Never	Rarely	Sometimes	Weekly	Daily	Yes	No
Solid stools^1^	85 (73.9%)	16 (13.9%)	7 (6.1%)	5 (4.3%)	2 (1.7%)		
Liquid stools^1^	17 (14.8%)	45 (39.1%)	27 (23.5%)	16 (13.9%)	10 (8.7%)		
Gas^1^	33 (28.9%)	20 (17.5%)	17 (14.9%)	22 (19.33%)	22 (19.3%)		
Lifestyle alterations^1^	61 (53%)	18 (15.7%)	18 (15.7%)	9 (7.8%)	9 (7.8%)		
Wears a pad^1*^	57 (49.1%)	18 (15.5%)	14 (12.1%)	8 (6.9%)	19 (16.4%)		57 (49.1%)
Use of medicine for constipation^2^						30 (25.9%)	86 (74.1%)
Ability to defer defecation for 15 minutes^2^						35 (30.2%)	81 (69.8%)
Ability to defer defecation for 10 minutes^3^						46 (37.9%)	70 (60.3%)
Ability to defer defecation for 5 minutes^3^						75 (64.7%)	41 (35.3%)
Ability to defer defecation for 1 minute^3^						99 (85.3%)	17 (14.7%)

Bowel urgency

Bowel urgency was prevalent and often severe: 41.6% of patients were unable to delay defecation for more than 15 minutes, as assessed by the Vaizey scale, although 28.9% of participants reported no FI (Table 3). Notably, 35.3% of FI participants could not hold their stools for over five minutes, and 14.7% could not hold them for over one minute.

**Figure 1:**
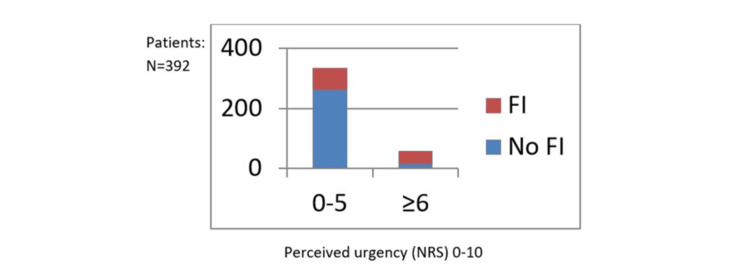
Urgency assessment based on NRS by fecal incontinence (FI) status (FI vs. no FI) NRS: numeric rating scale

Patients’ perceptions of urgency were also assessed using the urgency numeric rating scale (NRS). Severity scores on the urgency NRS were higher in patients with FI (37%) compared to the non-FI group (5.4%) (Figure 1).

FI concerns and communication

Less than half of participants (43.8%) considered FI an important topic, and among those patients, most (72.4%) reported having discussed it with their gastroenterologist. A little over a third of patients with FI (38.8%) said they considered it a risk before engaging in sexual intercourse.

Univariate analysis of FI risk factors

Factors suspected to affect FI were analyzed individually in a univariate analysis comparing participants reporting FI in the four previous weeks with those reporting no FI in the same period. FI was positively associated with being >50 years old (OR: 2.74; 95% CI: 1.57-3.90), having one or more vaginal deliveries (OR: 2.50; 95% CI: 1.32-4.71), disease duration > 15 years (OR: 2.04; 95% CI: 1.32-3.17), abdominal pain (OR: 2.33; 95% CI: 1.49-3.63), diarrhea (OR: 3.69; 95% CI: 2.29-5.96), blood in stools (OR: 3.2; 95% CI: 1.84-5.58), night bowel movements (OR: 4.46; 95% CI: 2.68-7.42), an SCCAI ≥ 3 (OR: 5.88; 95% CI: 2.66-13.03), and one or more flares in the current year (OR: 1.76; 95% CI: 1.12-2.75). Moreover, rectum involvement (OR: 1.57; 95% CI: 1.02-2.44), a history of anal fistula (OR: 2.80; 95% CI: 1.70-4.60), and having had two or more different therapies were equally associated with FI (OR: 1.79; 95% CI: 1.12-2.86) (Table 4).

**Table 4:** Risk factors associated with fecal incontinence (FI) in IBD patients: univariate and multivariate analyses Multivariate models adjusted for age, gender, and disease duration *Analysis for females only IBD: inflammatory bowel disease; OR: odds ratio; CI: confidence interval; UC: ulcerative colitis; CD: Crohn’s disease

Variable	Total (n = 392)	FI (n = 116)	No FI (n = 276)	Univariate OR	Lower CI	Upper CI	p-value	Multivariate OR	Lower CI	Upper CI	p-value
Age											
≤50	270 (68.9%)	64 (54.7%)	206 (74.9%)								
>50	122 (31.1%)	52 (45.3%)	70 (25.1%)	2.74	1.57	3.90	<0.001				
Sex											
Male	194 (49.5%)	55 (47%)	139 (50.4%)	1.15	0.75	1.78	0.522				
Female	198 (50.5%)	61 (53%)	137 (49.6%)								
Vaginal delivery*											
Never	127 (66.1%)	31 (51.7%)	96 (72.7%)								
Once or more	65 (33.9%)	29 (48.3%)	36 (27.3%)	2.50	1.32	4.71	0.005				
Smoker											
Yes	87 (22.2%)	33 (28.2%)	54 (19.6%)	1.61	0.98	2.65	0.063				
No	305 (77.8%)	84 (71.8%)	221 (80.4%)								
Disease type											
UC	138 (35.9%)	42 (36.2%)	96 (35.8%)	0.98	0.63	1.55	0.942				
CD	246 (64.1%)	74 (63.8%)	172 (64.2%)								
Disease duration											
≤15 years	229 (58.4%)	54 (46.2%)	175 (63.6%)								
>15 years	163 (41.6%)	63 (53.8%)	100 (36.4%)	2.04	1.32	3.17	0.001				
Rectum involved								1.60	1.01	2.49	0.045
Yes	195 (50.3%)	68 (58.1%)	127 (46.9%)	1.58	1.02	2.44	0.042				
No	193 (49.7%)	49 (41.9%)	144 (53.1%)								
History of anal fistula (not active)								2.51	1.48	4.26	<0.001
Yes	85 (21.9%)	41 (35%)	44 (16.2%)	2.80	1.70	4.60	<0.001				
No	304 (78.1%)	76 (65%)	228 (83.8%)								
Abdominal pain								2.56	1.60	4.09	<0.001
Yes	204 (52.2%)	44 (37.6%)	160 (58.4%)	2.33	1.49	3.63	<0.001				
No	187 (47.8%)	73 (62.4%)	114 (41.6%)								
Daily bowel movements								4.50	2.79	7.26	<0.001
0–2.9	264 (67.3%)	50 (42.7%)	214 (77.8%)								
≥3	128 (32.7%)	67 (57.3%)	61 (22.2%)	4.70	2.96	7.48	<0.001				
Night bowel movements								3.98	2.36	6.71	<0.001
Yes	83 (21.2%)	47 (40.2%)	36 (13.1%)	4.46	2.68	7.42	<0.001				
No	309 (78.8%)	70 (59.8%)	239 (86.9%)								
Arthropathy								1.83	1.13	2.98	0.015
Yes	107 (27.3%)	43 (11%)	64 (16.3%)	1.92	1.20	3.06	0.007				
No	285 (72.7%)	74 (18.9%)	211 (53.8%)								

EIMs were also associated with FI (OR: 1.18; 95% CI: 1.12-2.75), particularly arthropathy (OR: 1.92; 95% CI: 1.20-3.06). In subgroup analysis, FI was strongly associated with disease activity in UC patients (SCCAI ≥ 3: OR: 5.88; 95% CI: 2.66-13.03). In the CD cohort, however, the association with active disease (sCDAI) did not reach statistical significance (OR: 1.79; 95% CI: 0.88-3.62). FI was reported by 23.5% of CD and 12.3% of UC patients in clinical remission.

FI adversely affected general well-being, lowering QoL (OR: 2.56; 95% CI: 1.63-4.03). Depression affected 45 respondents (11.5%) in the global cohort. Among patients with FI, both depression (OR: 2.29; 95% CI: 1.22-4.3) and anxiety (OR: 1.76; 95% CI: 1.00-3.04) were significantly more frequent. Overall, nearly a third (31.4%) of patients reported experiencing poor well-being (Table 5).

**Table 5:** Association of self-reported depression, anxiety, and quality of life (QoL) with fecal incontinence (FI) in IBD patients Multivariate models adjusted for age, gender, and disease duration IBD: inflammatory bowel disease; OR: odds ratio; CI: confidence interval; WHO: World Health Organization

		FI, n (%)		Univariate analysis				Multivariate analysis				
Variable		Yes	No	Total n (%)	OR	Lower CI	Upper CI	p-value	Adjusted OR	Lower CI	Upper CI	p-value
Depression	Y	21 (17.9)	24 (8.7)	45 (11.5%)	2.29	1.22	4.30	0.01	2.43	1.27	4.68	0.008
	N	96 (82.1)	251 (91.3)	347 (88.5%)								
Anxiety	Y	27 (23.1)	40 (14.5)	67 (17.1%)	1.76	1.00	3.04	0.042	1.88	1.07	3.30	0.029
	N	90 (76.9)	235 (85.5)	325 (82.9%)								
WHO-5 well-being index (QoL)	≤50 (poor)	54 (46.2)	69 (25.1)	123 (31.4%)	2.56	1.63	4.03	<0.001	2.64	1.65	4.24	<0.001
	>50	63 (53.8)	206 (74.9)	269 (62.6%)								
Self-reported well-being	Good	52 (44.4%)	185 (67.5%)	237 (60.6%)								
	Below “good”	65 (55.6%)	89 (32.5%)	154 (39.4%)	2.60	1.67	4.05	<0.001	2.61	1.65	4.14	0.06

Multivariate analysis of FI risk factors

Multivariate logistic regression identified several independent predictors of FI: abdominal pain (OR: 2.56; 95% CI: 1.60-4.09), diarrhea (OR: 3.70; 95% CI: 2.27-6.04), blood in stools (OR: 3.33; 95% CI: 1.88-5.90), night bowel movements (OR: 3.98; 95% CI: 2.36-6.71), an SCCAI ≥ 3 (OR: 6.06; 95% CI: 2.62-14.02), one or more flares in the current year (OR: 1.90; 95% CI: 1.19-3.02), rectal involvement (OR: 1.60; 95% CI: 1.01-2.49), a history of anal fistula (OR: 2.51; 95% CI: 1.48-4.26), the presence of one or more EIM (OR: 1.64; 95% CI: 1.03-2.61), and arthropathy (OR: 1.83; 95% CI: 1.13-2.98). Psychological burden remained significantly elevated in patients with FI, reporting reduced QoL (OR: 2.64; 95% CI: 1.65-4.24), increased depression (OR: 2.43; 95% CI: 1.27-4.68), and anxiety (OR: 1.88; 95% CI: 1.07-3.30).

## Discussion

In this study of IBD patients receiving regular care, almost one-third (29.5%) said they experienced FI in the last four weeks, showing that FI is a common but often overlooked issue for people with IBD. This prevalence exceeds earlier European estimates using similar timeframes, such as Vollebregt et al. [18] (20%) and Kurt et al. [9] (16.4%). The higher rate may reflect the disease severity in our cohort, where over 90% were treated with biologics, putting them at a higher risk for FI.

FI was significantly associated with classical disease activity markers: diarrhea, bloody stools, nocturnal bowel movements, abdominal pain, and urgency. This supports earlier research suggesting that both active disease and high disease activity significantly contribute to FI [3,8,9,19].

Urgency was a key issue: over two-thirds of patients with FI (69.8%) could not wait more than 15 minutes to have a bowel movement, which is similar to a Swiss survey that found 69.7% of people felt an urgent need every day [20]. These findings support recent information highlighting the connection between urgency and incontinence in UC and suggest that checking for urgency should be a key part of medical evaluations [21]. Despite healthcare professionals’ awareness of the severity of urgency, recent studies have shown that its burden, notably on CD patients, is often underestimated [22-24]. Measuring varying levels of urgency based on delays of 10, five, and one minute offered more granularity in understanding its severity; 35.3% of IBD patients with FI could not hold their stools for more than five minutes, illustrating the severity and unpredictability of symptoms.

Being over 50 years old and having the disease for more than 15 years were linked to FI on their own, likely due to ongoing tissue damage and age-related issues with the anal area. No association was found with sex or disease type, consistent with existing findings [3,25].

Active disease in UC, measured by SCCAI ≥ 3, was the strongest independent predictor of FI. This reinforces the notion that active inflammation is a significant contributor to FI in patients with IBD, aligning with existing literature [3,4,8,9]. However, a significant number of patients with no active disease still experienced FI-23.5% in CD and 12.3% in UC-indicating that inflammation is not the only reason for ongoing symptoms. This observation mirrors recent findings that revealed substantial urgency and continence burden even in remission [22,23,26].

Contrary to existing literature, no notable link was found between FI and disease activity in CD patients [4,8,9]. It is important to note that, owing to the survey type, the sCDAI was calculated using data from just one day rather than from a seven-day collection, possibly leading to an underestimation of disease severity.

A particularly novel finding in our study is the significant association between a history of anal fistula and FI, even in the absence of active fistula. While previous literature emphasizes current fistulizing disease, our results suggest that longstanding damage may lead to irreversible changes in continence mechanisms.

Similarly, previous reports have not documented the association between EIMs, especially arthropathy, and FI. This may reflect more aggressive systemic disease or shared neuroinflammatory pathways [26]. Thus, it underscores the necessity for a holistic strategy in IBD management. The presence of EIMs should prompt clinicians to assess continence symptoms.

In addition to its physical burden, FI was strongly linked to lower QoL, which is further exacerbated by notably higher instances of depression and anxiety in these individuals. These findings support recent qualitative research on the psychological burden of bowel symptoms in IBD [2,21,24]. Healthcare providers must not only address the physical aspects of IBD but also actively discuss emotional well-being and mental health with patients. By tackling these issues, we can significantly enhance the patients’ overall QoL.

Some limitations must be acknowledged. This study was intentionally designed as a real-world, patient-centered assessment. The use of anonymous questionnaires helped participants share sensitive issues like FI more openly, but it also meant we could not gather detailed clinical data, specific biomarkers such as fecal calprotectin, or other related conditions like irritable bowel syndrome (IBS). While IBS may contribute to FI, evaluating its presence was outside the scope of this study. FI was assessed using the Wexner and Vaizey scores, which measure incontinence over the past four weeks rather than following the stricter Rome IV definition. This broader approach may overestimate prevalence but offers advantages in IBD by capturing urgency, using validated and reliable tools, and remaining brief and patient-friendly. We used these scores as they are validated in IBD and allow direct comparison with previous studies. The Pelvic Floor Disorders Consortium has recommended the use of the IMPACT tool-a more comprehensive and concise measure that integrates elements from Wexner and Vaizey while improving sensitivity to urgency symptoms. While such broader instruments could holistically assess pelvic floor dysfunction and be innovative, they lack IBD validation; a hybrid approach combining established severity scales with selected IMPACT domains could be valuable for future research [27]. Moreover, as a cross-sectional study, our design identifies associations but does not establish causality, and reliance on self-reported data might introduce recall bias. Here, the 50.3% rate of rectal involvement likely underestimates the true prevalence. Finally, we acknowledge the absence of a universally accepted diagnostic algorithm for FI that integrates symptom severity, urgency, psychological impact, and underlying etiology. Future research aimed at better understanding the pathophysiology could combine subjective severity scores with objective assessments, such as biochemical markers like fecal calprotectin and pelvic floor imaging modalities such as rectoanal endosonography. Our study focused on estimating FI prevalence and associated factors in a real-life clinical setting rather than defining underlying mechanisms, but more comprehensive approaches would be warranted in future work. However, our study stands out as one of the few in Europe that employs validated patient-reported instruments to evaluate FI, urgency, and psychosocial burden in IBD.

## Conclusions

In our survey, 29.5% of patients with IBD reported having FI, confirming its high prevalence and clinical relevance. FI was associated with older age, prior vaginal delivery, longer disease duration, abdominal pain, diarrhea, rectal bleeding, nocturnal bowel movements, and active disease (SCCAI ≥ 3). Importantly, having a history of anal fistula and extraintestinal symptoms, especially joint pain, were found to be newly identified factors.

Patients with FI frequently experienced severe urgency; nearly 15% were unable to delay defecation by more than one minute. FI was also linked to reduced QoL, along with elevated rates of anxiety and depression. These findings point out the value of holistic management strategies in IBD and call for routine screening of FI and urgency in clinical practice. Practically, healthcare providers should be aware of these risk factors and adopt a comprehensive, multidisciplinary approach that addresses both physical symptoms and psychological well-being. Future research is required to better understand the mechanisms underlying FI in IBD and identify practical, evidence-based strategies to reduce its burden and enhance patient QoL.

## Appendices

Supplemental material 1 

**Table 6:** Questionnaire (French version)

QUESTIONNAIRE																																
Chers patients, Merci de prendre votre précieux temps pour remplir ce questionnaire sur les Maladies inflammatoires chroniques de l’intestin (=MICI) et l'incontinence fécale. Nous souhaitons en savoir plus sur ce sujet afin d'améliorer notre prise en charge globale. Ce thème est souvent tabou, pourtant il peut être très gênant au quotidien et altérer considérablement la qualité de vie d'une personne. Toutes les réponses recueillies sont anonymes et utilisées pour une thèse médicale. Les résultats seront ensuite publiés et un retour d'information pourra vous être donné, si vous le souhaitez. Les questions sont sur votre état surles 7 derniers jours																																
A propos de vous																																
Sexe:																																
Féminin																		Masculin														
Pour les femmes: Avez-vous eu des accouchements par voie vaginale ( pas césarienne)?																																
0						1										2					3								>3			
Année de naissance :																																
____																																
Année du diagnostic de la MICI :																																
____																																
Fumeur :																																
Oui																		Non														
A propos de votre maladie																																
Maladie:																																
Colite ulcéreuse									Morbus Crohn														Pas clair									
Atteinte du rectum (la fin de l’intestin)																																
Oui																		Non														
Avez-vous déjà eu une fistule anale ?																																
Oui																		Non														
à Si oui:																																
_____ est-elle actuellement active ?																																
Oui																		Non														
____ avez-vous été opéré (Seton/drainage)																																
Oui																		Non														
Avez-vous des douleurs abdominales ?																																
Aucune							Légère											Moyenne									Forte					
Avez-vous des traces de sang dans les selles ?																																
Aucune							Légère											Modérée									Seulement du sang					
Nombre de selles liquides ou très molles par jour																																
____																																
Combien de selles avez-vous pendant la journée ?																																
____																																
Combien de selles avez-vous la nuit ?																																
____																																
Combien de poussées avez-vous eu cette année ?																																
0										1-2														≥3								
Avez-vous déjà eu un traitement biologique ? (p.ex, Remicade®, Inflectra, Entyvio®, Stelara®, Xeljanz®, Zeposia®, Humira®, Cimzia®, Simponi®)																																
Oui																		Non														
Quel traitement avez-vous actuellement ?																																
____																																
Combien de thérapies différentes avez-vous eu ?																																
0							1											2-3									≥4					
Avez-vous eu des douleurs articulaires qui étaient plus graves au repos qu'en mouvement ?																																
Oui																		Non														
Vos articulations sont-elles rouges ou gonflées ?																																
Oui																		Non														
Avez-vous été réveillé par des douleurs articulaires ?																																
Oui																		Non														
Avez-vous une maladie de peau diagnostiquée comme étant un érythème noueux ?																																
Oui																		Non														
Avez-vous une maladie de la peau diagnostiquée comme étant un pyoderma gangrenosum ?																																
Oui																		Non														
Avez-vous une maladie oculaire diagnostiquée comme étant une uvéite?																																
Oui																		Non														
A propos de l'incontinence fécale/Urgence= besoin d'aller à la selle L'incontinence fécale signifie que vous ne pouvez pas contrôler vos selles et qu'un peu de selles pourrait par exemple se retrouver dans vos sous-vêtements. L'urgence est définie comme le besoin impérieux d'aller à la selle.																																
L'incontinence/l’urgence sont-ils des sujets important pour vous ?																																
Oui											Non														Pas d’opinion							
En parlez-vous ouvertement avec votre médecin ?																																
Oui																		Non														
Y pensez-vous consciemment avant d'avoir des rapports sexuels ?																																
Oui																		Non														
Définition pour les questions suivantes : 0= Jamais : aucun événement (par exemple incontinence fécale) au cours des 4 dernières semaines 1=Rarement : 1 événement au cours des 4 dernières semaines ; 2= Parfois : >1 événement au cours des 4 dernières semaines, mais <1 par semaine ; 3=Hebdomadaire : 1 événement ou plus par semaine, mais <1 par jour ; 4=Quotidiennement : 1 événement ou plus par jour.																																
Souffrez-vous d'incontinence ? -Si Non, (Jamais (0)), veuillez passer directement à la question ***																																
0					1										2					3								4				
Incontinence pendant la journée																																
0					1										2					3								4				
Incontinence nocturne																																
0					1										2					3								4				
Incontinence de selles solides																																
0					1										2					3								4				
Incontinence de selles liquides																																
0					1										2					3								4				
Incontinence de gazs																																
0					1										2					3								4				
Selon le nombre d'incontinence fécale, je me sens :																																
Très mal		Mal									OK							Bien							Très bien						Excellent	
question *** : Evitez-vous de sortir de chez vous à cause de l'incontinence/de la peur d’épisodes d’incontinence ?																																
Jamais					1x/Mois										2–3 x/Mois					1x/semaine								Tout les jours				
Avez-vous peur de faire dans votre pantalon lorsque vous êtes à l'extérieur ?																																
0					1										2					3								4				
Portez-vous des serviettes hygiéniques lorsque vous sortez ?																																
0					1										2					3								4				
Pouvez-vous retenir vos selles pendant <1 min ?																																
Oui																		Non														
Pouvez-vous retenir vos selles pendant 5 min ?																																
Oui																		Non														
Pouvez-vous retenir vos selles pendant 10 min ?																																
Oui																		Non														
Pouvez-vous retenir vos selles pendant 15 min ?																																
Oui																		Non														
Prenez-vous des médicaments pour vous constiper ? (par exemple Imodium)																																
Oui																		Non														
Quelle était l'urgence de votre envie d'aller à la selle au cours des dernières 24 heures ? 0 : (pas d’urgence) 10 :(selles très urgente)																																
0	1			2				3						4			5		6			7				8				9		10
À propos de la dépression/de l'anxiété																																
L'incontinence/l'urgence a-t-elle entraîné une dépression ?																																
Ne souffre pas d'incontinence/d'urgence												Oui													Non							
Souffrez-vous de dépression ?																																
Oui																		Non														
Souffrez-vous d'anxiété ?																																
Oui																		Non														
A propos de votre bien-être																																
Quel est votre bien être général au quotidien ? Bien-être sur 7 jours																																
Bon					Légèrement en dessous de la moyenne										Mauvais					Très mauvais								Catastrophique				
Veuillez indiquer, pour chacune des cinq affirmations, laquelle se rapproche le plus de ce que vous avez ressenti au cours des deux dernières semaines. Notez que le chiffre est proportionnel au bien-être. Définition : 0= Jamais 1= De temps en temps 2= Moins de la moitié du temps 3= Plus de la moitié du temps 4= La plupart du temps 5= Tout le temps																																
Au cours des deux dernières semaines ... ... Je me suis senti(e) bien et de bonne humeur																																
0			1										2					3							4						5	
Au cours des deux dernières semaines ... … Je me suis senti(e) calme et tranquille																																
0			1										2					3							4						5	
Au cours des deux dernières semaines ... ... …Je me suis senti(e) plein(e) d’énergie et vigoureux(se)																																
0			1										2					3							4						5	
Au cours des deux dernières semaines ... …Je me suis réveillé(e) en me sentant frais(che) et dispos(e)																																
0			1										2					3							4						5	
Au cours des deux dernières semaines ... …Ma vie quotidienne a été remplie de choses intéressantes																																
0			1										2					3							4						5	
Merci beaucoup pour votre participation ! Si vous avez un commentaire/conseil concernant ce thème pour l'équipe (infirmières/médecins), n'hésitez pas à le partager ici.																																
____																																

Supplemental material 2 

**Table 7:** Questionnaire (German version)

FRAGEBOGEN																																
Liebe Patientinnen und Patienten, Vielen Dank, dass Sie sich die Zeit nehmen, diesen Fragebogen über chronisch entzündliche Darmerkrankungen (CED) und Stuhlinkontinenz auszufüllen.Stuhlinkontinenz bedeutet, dass Sie Ihren Stuhl nicht kontrollieren können (Ihnen z.B. etwas Stuhl in die Unterwäsche gehen könnte). Um die Betreuung global zu verbessern, möchtenwir mehr über dieses Thema erfahren. Dieses Thema ist oft Tabu, kann im täglichen Leben sehr unangenehm sein und die Lebensqualität drastisch beeinträchtigen. Alle gesammelten Antworten werden anonymisiert und für eine Doktorarbeit verwendet. Die Ergebnisse werden anschliessend veröffentlicht und auf Wunsch kann Ihnen ein Feedback gegeben werden. Die Fragen beziehen sich auf Ihren Zustandder letzten 7 Tage																																
Über Sie																																
Geschlecht:																																
weiblich																		männlich														
Für Frauen: wie viele vaginale Geburten hatten Sie bisher?																																
0						1										2					3								>3			
Jahrgang:																																
____																																
Jahr der Diagnose der CED:																																
____																																
Raucher:																																
Ja																		Nein														
Über die Krankheit																																
Krankheit:																																
Colitis ulcerosa									Morbus Crohn														unklar									
Befall des Rektums (Enddarm):																																
Ja																		Nein														
Haben Sie schon eine Analfistel gehabt?																																
Ja																		Nein														
à Falls ja,																																
_____ ist sie momentan aktiv?																																
Ja																		Nein														
_____ wurden Sie operiert (Seton/Drainage)?																																
Ja																		Nein														
Bauchschmerzen?																																
Keine							Leicht											Mässig									Stark					
Blut im Stuhl?																																
Keine							Leicht											Mässig									Nur Blut					
Anzahl der flüssigen oder sehr weichen Stuhlgänge pro Tag																																
____																																
Wie viele Stühle haben Sie tagsüber?																																
____																																
Wie viele Stühle haben Sie nachts?																																
____																																
Wie viele Schübe haben Sie dieses Jahr gehabt?																																
0										1-2														≥3								
Haben Sie schon eine Biologika-Therapie gemacht? (e.g., Remicade®, Inflectra, Entyvio®, Stelara®, Xeljanz®, Zeposia®, Humira®, Cimzia®, Simponi®)																																
Ja																		Nein														
Welche Therapie machen Sie im Moment?																																
____																																
Wie viele unterschiedliche Therapien haben Sie gehabt?																																
0							1											2-3									≥4					
Hatten Sie Gelenksschmerzen,die im Ruhezustand schlimmer waren als in Bewegung?																																
Ja																		Nein														
Sind Ihre Gelenke rot oder geschwollen?																																
Ja																		Nein														
Wurden Sie von Gelenksschmerzen geweckt?																																
Ja																		Nein														
Haben Sie eine Hauterkrankung, dieals Erythema nodosum diagnostiziert wurde?																																
Ja																		Nein														
Haben Sie eine Hauterkrankung, die als Pyoderma diagnostiziert wurde?																																
Ja																		Nein														
Haben Sie eine Augenerkrankung, bei der eine Uveitis diagnostiziert wurde?																																
Ja																		Nein														
Über Stuhlinkontinenz/Urgency= Stuhldrang Stuhlinkontinenz bedeutet dass Sie Ihren Stuhl nicht kontrollieren können und Ihnen zB etwas Stuhl in die Unterwäsche gelangen könnte. Urgency ist auch als Stuhldrang definiert es beschreibt der Drang zur Stuhlentleerung																																
Ist Inkontinenz/Stuhldrang ein wichtiges Thema für Sie?																																
Ja											Nein														Keine Meinung							
Sprechen Sie mit Ihrem Arzt offen darüber?																																
Ja																		Nein														
Denken Sie vor Geschlechtsverkehr bewusst darüber nach?																																
Ja																		Nein														
Definition für folgende Fragen: 0= Nie: kein Ereignis (zB Stuhlinkontinez) in den letzten 4 Wochen; 1=Selten: 1 Ereignis in den letzten 4 Wochen; 2= Manchmal: >1 Ereignis in den letzten 4 Wochen, aber <1 pro Woche; 3=Wöchentlich: 1 oder mehr Ereignis pro Woche, aber <1 pro Tag; 4=Täglich: 1 oder mehr Ereignis pro Tag																																
Leiden Sie an Inkontinenz? àFalls Nie (0), bitte direkt zu Frage ***																																
0					1										2					3								4				
Inkontinenztagsüber																																
0					1										2					3								4				
Inkontinenz nachts																																
0					1										2					3								4				
Inkontinenz bei festem Stuhl																																
0					1										2					3								4				
Inkontinenz bei flüssigem Stuhl																																
0					1										2					3								4				
Inkontinenz für Luft?																																
0					1										2					3								4				
Je nach Anzahl der Stuhlinkontinenz, ich fühle mich:																																
Sehr schlecht		Schlecht									In Ordnung							Gut							Sehr gut						Ausgezeichnet	
Frage *** Vermeiden Sie es das Haus zu verlassen aufgrund der Inkontinenz/Angst davor?																																
Nie					1x/Monat										2–3 mal/Monat					1x/Woche								Täglich				
Haben Sie Angst, in die Hose zu machen wenn Sie draussen sind?																																
0					1										2					3								4				
Tragen Sie Binden,wenn Sie nach draussen gehen?																																
0					1										2					3								4				
Können Sie während<1 minute Stuhl halten?																																
Ja																		Nein														
Können Sie während 5 minuten Stuhl halten?																																
Ja																		Nein														
Können Sie während 10 minuten Stuhl halten?																																
Ja																		Nein														
Können Sie während 15 minuten Stuhl halten?																																
Ja																		Nein														
Nehmen SieMedikamente zum Stopfen ein? (zB Imodium)																																
Ja																		Nein														
Wie stark war Ihr in den letzten 24 Stunden? 0:(Kein Stuhldrang) 10:(schlimmstmöglicher Stuhldrang)																																
0	1			2				3						4			5		6			7				8				9		10
Über Depression/Ängste																																
Führte die Inkontinenz/Urgency zu einer Depression?																																
Habe Keine Inkontinenz/Urgency												Ja													Nein							
Leiden Sie an einer Depression?																																
Ja																		Nein														
Leiden Sie an Angstzuständen?																																
Ja																		Nein														
Über Ihr Wohlbefinden																																
Allgemeines tägliches Wohlbefinden über 7 Tage																																
Gut					Etwas unter Durchschnitt										Schlecht					sehr schlecht								Furchtbar				
Die folgenden Aussagen betreffen Ihr Wohlbefinden in den letzten zwei Wochen. Bitte markieren Sie bei jeder Aussage die Rubrik, die Ihrer Meinung nach am besten beschreibt, wie Sie sich in den letzten zwei Wochen gefühlt haben. Definition: 0= Zu keinem Zeitpunkt 1=Ab und zu 2= Etwas weniger als die Hälfte der Zeit 3=Etwas mehr als die Hälfte der Zeit 4=Meistens 5=Die ganze Zeit																																
In den letzten zwei Wochen … … war ich froh und guter Laune																																
0			1										2					3							4						5	
In den letzten zwei Wochen … … habe ich mich ruhig und entspannt gefühlt																																
0			1										2					3							4						5	
In den letzten zwei Wochen … ...habe ich mich energisch und aktiv gefühlt																																
0			1										2					3							4						5	
In den letzten zwei Wochen … … habe ich mich beim Aufwachen frisch und ausgeruht gefühlt																																
0			1										2					3							4						5	
In den letzten zwei Wochen … … war mein Alltag voller Dinge, die mich interessieren																																
0			1										2					3							4						5	
Vielen Dank für Ihre Teilnahme! Falls Sie ein Kommentar/Tipp bezüglich dieses Themas für das Team (Nurse/Ärzte) haben, können Sie das gerne hier mitteilen																																
____																																

Supplemental material 3 

**Table 8:** Questionnaire about fecal incontinence; English version, original in French and German

QUESTIONNAIRE																												
Dear patients, Thank you for taking the time to complete this questionnaire on inflammatory bowel diseases (IBDs) and fecal incontinence. Fecal incontinence means the inability to control bowel movements (for example, some stool might unintentionally soil your underwear). To improve care overall, we would like to learn more about this topic. Although it is often a taboo subject, it can cause significant discomfort in daily life and drastically reduce quality of life. All responses are anonymous and will be used for a doctoral thesis. The results will be published, and feedback can be provided upon request.																												
About you																												
Gender:																												
Female															Male													
For females, how many vaginal births have you had?																												
0			1										2					3								>3		
Year of birth:																												
____																												
Year of IBD diagnosis:																												
____																												
Smoker:																												
Yes															No													
About the disease																												
Type of disease:																												
Ulcerative colitis						Crohn’s disease														Unclear								
Rectal involvement (end of large bowel):																												
Yes															No													
Have you ever had an anal fistula?																												
Yes															No													
→ If yes:																												
Is it currently active?																												
Yes															No													
Have you had surgery (seton/drainage)?																												
Yes															No													
Abdominal pain in the last 7 days																												
None				Mild											Moderate									Severe				
Blood in stool in the last 7 days																												
None				Mild											Moderate									Only blood				
Number of liquid or very soft stools per day in the last 7 days																												
____																												
How many bowel movements during the day in the last 7 days?																												
____																												
How many bowel movements during the night in the last 7 days?																												
____																												
How many flare-ups have you had this year?																												
0							1-2														≥3							
Have you ever received biological therapy? (e.g., Remicade^®^, Inflectra, Entyvio^®^, Stelara^®^, Xeljanz^®^, Zeposia^®^, Humira^®^, Cimzia^®^, Simponi^®^)																												
Yes															No													
Which therapy are you currently receiving?																												
____																												
How many different therapies have you received?																												
0				1											2-3									≥4				
Have you had joint pain that was worse at rest than during movement?																												
Yes															No													
Are your joints red or swollen?																												
Yes															No													
Have you been woken up by joint pain?																												
Yes															No													
Have you had a skin disease diagnosed as erythema nodosum?																												
Yes															No													
Have you had a skin disease diagnosed as pyoderma gangrenosum?																												
Yes															No													
Have you had an eye disease diagnosed as uveitis?																												
Yes															No													
About fecal incontinence/urgency																												
Fecal incontinence means the inability to control stool, for example, leading to soiling of underwear. Urgency is defined as the sudden, strong urge to defecate that is difficult to control																												
Is incontinence/urgency an important issue for you?																												
Yes								No														No opinion						
Do you speak openly with your doctor about it?																												
Yes															No													
Do you consciously think about it before sexual intercourse?																												
Yes															No													
Definitions for the following questions: 0 = never: no event in the last 4 weeks; 1 = rarely: 1 event in the last 4 weeks; 2 = sometimes: more than 1 event in the last 4 weeks, but less than once per week; 3 = weekly: 1 or more events per week, but less than once per day; 4 = daily: 1 or more events per day																												
Do you suffer from fecal incontinence? → If never (0), please skip to question ***																												
0			1									2					3								4			
Incontinence during the day																												
0			1									2					3								4			
Incontinence at night																												
0			1									2					3								4			
Incontinence with solid stool																												
0			1									2					3								4			
Incontinence with liquid stool																												
0			1									2					3								4			
Incontinence with gas (flatus)																												
0			1									2					3								4			
How do you feel about your incontinence?																												
Very bad	Bad							Okay							Good							Very good					Excellent	
Question ***: Do you avoid leaving the house due to incontinence or fear of it?																												
Never			Once a month									2–3 times/month					Once a week								Daily			
Are you afraid of having an accident (soiling) when outside?																												
0			1									2					3								4			
Do you wear pads when going out?																												
0			1									2					3								4			
Can you hold your stool for less than 1 minute?																												
Yes															No													
Can you hold your stool for 5 minutes?																												
Yes															No													
Can you hold your stool for 10 minutes?																												
Yes															No													
Can you hold your stool for 15 minutes?																												
Yes															No													
Do you take antidiarrheal medication (e.g., Imodium)?																												
Yes															No													
How severe was your urge to defecate in the past 24 hours? (0 = no urgency; 10 = worst imaginable urgency)																												
0	1		2		3						4			5		6			7				8			9		10
About depression/anxiety																												
Has your incontinence or urgency led to depression?																												
I don’t have incontinence or urgency									Yes													No						
Do you suffer from depression?																												
Yes															No													
Do you suffer from anxiety?																												
Yes															No													
About your well-being																												
Overall daily well-being over the last 7 days																												
Good			Slightly below average									Poor					Very poor								Terrible			
The following statements refer to how you felt during the last 2 weeks. Please mark the box that best reflects how you felt. Definitions: 0 = at no time; 1 = occasionally; 2 = less than half of the time; 3 = more than half of the time; 4 = mostly; 5 = all of the time																												
In the last two weeks... … I felt cheerful and in good spirits																												
0		1								2					3							4					5	
In the last two weeks... … I felt calm and relaxed																												
0		1								2					3							4					5	
In the last two weeks... … I felt energetic and active																												
0		1								2					3							4					5	
In the last two weeks... … I felt fresh and rested when waking up																												
0		1								2					3							4					5	
In the last two weeks... … My daily life was filled with things that interest me																												
0		1								2					3							4					5	
Thank you very much for your participation! If you have any comments or suggestions for our team (nurses/doctors), feel free to share them here.																												
____																												
